# AI-Driven Generation of Post-Contrast T1 and ECV Maps from Native T1 Map in Cardiac MRI

**DOI:** 10.3390/diagnostics16152308

**Published:** 2026-07-23

**Authors:** Young Jung Yang, Ga Hyeon Kim, Yoon-Chul Kim, Young Jin Kim

**Affiliations:** 1 Phantomics, Inc., Magokseoro 152, Gangseo-gu, Seoul 07788, Republic of Korea; yjyang@phantomics.io; 2 Department of Digital Healthcare, College of Health Sciences, Yonsei University, Wonju 26493, Republic of Korea; gghh2032@naver.com; 3 Department of Radiology, Research Institute of Radiological Science, Severance Hospital, Yonsei University College of Medicine, 50-1 Yonsei-ro, Seodaemun-gu, Seoul 03722, Republic of Korea; dryj@yuhs.ac

**Keywords:** cardiovascular magnetic resonance imaging, deep learning, T1 mapping, extracellular volume fraction, medical image analysis

## Abstract

**Background/Objectives**: This study aimed to develop an artificial intelligence-based method for generating virtual post-contrast T1 maps and extracellular volume (ECV) maps from native T1 maps and to evaluate its performance. **Methods**: The proposed method was based on a modified self-consistent recursive diffusion bridge framework to generate virtual post-contrast T1 maps from native T1 maps. Cardiac magnetic resonance (CMR) data were collected from consecutive patients with suspected myocardial disease. A total of 813 well-registered image slices were selected for model development and evaluation. On an unseen test set of native T1 maps, the trained model generated virtual post-contrast T1 maps, which were subsequently combined with the corresponding native T1 maps to compute ECV maps. **Results**: The myocardial T1 values derived from the reference and virtual post-contrast T1 maps revealed similar distributions, although a systematic offset between the distribution peaks was observed. Following ECV transformation, this offset was substantially reduced. In the held-out test cohort, the virtual myocardial ECV showed acceptable agreement with the reference ECV, achieving a mean root mean square error (RMSE) of 3.05%, despite noticeable slice-to-slice variability (R^2^ = 0.585; Bland–Altman 95% limits of agreement, −5.98% to +6.06%). **Conclusions**: The proposed method enabled the generation of post-contrast T1 and ECV maps directly from native T1 maps without the administration of gadolinium-based contrast agents during CMR. These findings suggest that the proposed approach represents a promising contrast-free, non-invasive alternative for myocardial tissue characterization, with the potential to reduce examination costs, eliminate contrast-agent-related risks, and improve patient safety.

## 1. Introduction

The extent and distribution of myocardial fibrosis can be quantitatively assessed using late gadolinium enhancement (LGE) imaging or T1 mapping in cardiac magnetic resonance imaging (MRI) [1]. LGE imaging is effective for detecting focal myocardial lesions, such as infarction, in patients with coronary artery disease [2]. In contrast, extracellular volume (ECV) mapping derived from T1 mapping provides quantitative information for assessing diffuse myocardial fibrosis, which is commonly observed in patients with non-ischemic heart disease [3].

Recent advances in generative artificial intelligence (AI) have accelerated research in computer vision and medical image analysis, including cardiac MRI. Recent research studies in cardiac imaging have explored generative AI for image enhancement [4], automatic infarct segmentation from cardiac cine MRI [5,6,7], and virtual LGE image synthesis through image-to-image translation [8,9,10,11]. The ability to generate high fidelity virtual LGE images has the potential to reduce scan time and eliminate the need for gadolinium-based contrast agent administration, particularly in patients with contraindications to contrast agents.

Beyond virtual LGE image synthesis, recent studies have also investigated AI-based generation of synthetic T1-weighted images and T1 maps. A generative adversarial network (GAN) was trained using a single inversion-time image to generate synthetic images at different inversion times [12]. Synthetic T1-weighted images have also been used to train neural networks for myocardial segmentation [13]. Post-contrast T1 images were generated by utilizing GANs [14]. Despite these promising results, GANs are often difficult to train and are susceptible to mode collapse. More recently, diffusion models have emerged as a powerful alternative for generative image synthesis [15,16]. They have been successfully applied to image augmentation in cardiac cine MRI [17] and cross-modality image translation in a variety of applications, including MRI-to-CT translation of brain images [18,19].

Conventional denoising diffusion models gradually add noise to clean images during the forward diffusion process and reconstruct images from pure noise during the reverse process. Although these models can generate realistic images sampled from the training data distribution, they often struggle to preserve the high structural fidelity required for image-to-image translation tasks, such as image restoration, deblurring, super-resolution, and image inpainting. To address this limitation, diffusion bridge models have been proposed to explicitly learn the transformation between source and target image distributions [20,21,22,23,24]. Rather than generating images solely from random noise, diffusion bridges progressively generate intermediate samples that interpolate between the source and target images, thereby improving translation fidelity.

Recently, Arslan et al. proposed the Self-consistent Recursive Diffusion Bridge (SelfRDB), which formulates cross-modality image translation as a stochastic reverse-diffusion process with recursive self-consistency constraints [18]. They demonstrated its effectiveness for translating among multiple MRI sequences as well as MRI-to-CT translation of axial brain images. To the best of our knowledge, this is the first study to apply SelfRDB for synthesizing post-contrast T1 maps in cardiac MRI. The generated post-contrast T1 maps are subsequently used to derive synthetic ECV maps. We evaluate the proposed framework using paired native and post-contrast T1 maps acquired from patients with suspected myocardial disease and assess both the quality of the synthesized post-contrast T1 maps and the accuracy of the resulting ECV maps.

## 2. Materials and Methods

**Figure 1:**
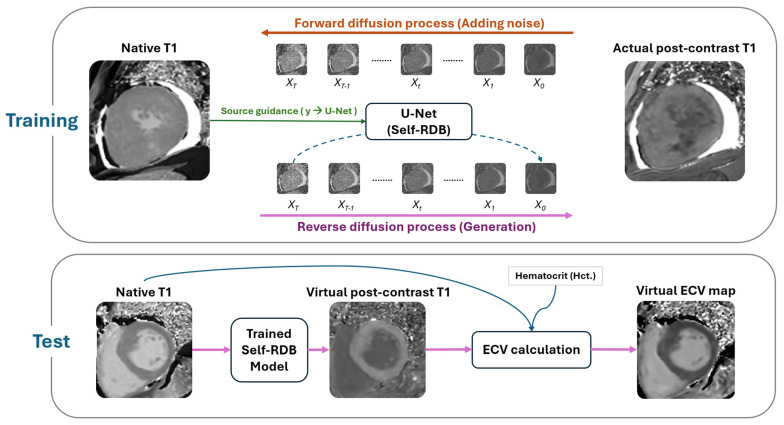
A flowchart of our study.

An overview of the proposed framework is presented in Figure 1. The proposed diffusion model takes a native T1 map as input and generates a corresponding post-contrast T1 map through image-to-image translation. The generated post-contrast T1 map is subsequently combined with the corresponding native T1 map to compute an ECV map.

### 2.1. Dataset

We retrospectively collected data from consecutive patients with suspected myocardial disease who underwent cardiac magnetic resonance (CMR) imaging at a tertiary hospital between 2018 and 2020. The study was approved by the institutional review board of Yonsei University Health System (No. 4-2024-1483).

Native and post-contrast T1 mapping was performed using a modified Look-Locker inversion recovery (MOLLI) sequence on a 3.0-T MRI scanner (MAGNETOM Prisma Fit, Siemens Healthineers, Erlangen, Germany). Native MOLLI images were acquired using the following parameters: repetition time/echo time (TR/TE), 264.56/1.09 ms; flip angle, 35°; slice thickness, 8 mm; field-of-view (FOV), 380 × 315 mm; image matrix, 512 × 424; and 24 images across eight inversion times (TIs) per set, with a TI range of 102–3957 ms. Post-contrast MOLLI images were acquired using the same imaging geometry, but with a TR of 394.16 ms and 27 images acquired across nine TIs per set, with a TI range of 112–2950 ms, optimized for the shorter post-contrast T1 values.

Data from a total of 271 patients with hypertrophic cardiomyopathy were included in this study. For each patient, native and post-contrast T1 maps were available at the apical, midventricular, and basal short-axis levels. Thus, three slices were available per patient, resulting in a total of 813 slices, as summarized in Table 1.

### 2.2. Image Preprocessing

All native and post-contrast T1 maps were exported from the picture archiving and communication system (PACS) as quantitative parametric images. The left ventricular (LV) epicardial and endocardial contours were automatically segmented using the two-dimensional implementation of nnU-Net v2 [25,26]. Separate segmentation models were trained for native T1 maps using 2111 training cases and for post-contrast T1 maps using 2107 training cases. For each subject, three adjacent short-axis slices were provided as input during model inference. The predicted myocardial mask was refined by retaining only the largest connected component. Epicardial and endocardial contours were extracted using morphology-based post-processing. A bounding box centered on the epicardial mask was isotropically expanded by a factor of 1.7 to define the cardiac region of interest. Native T1 images were cropped according to the epicardial contours obtained from the native T1 images, whereas post-contrast T1 images were cropped according to the corresponding post-contrast epicardial contours. All cropped images were subsequently resized to 224 × 224 pixels using bicubic interpolation.

For intensity preprocessing, native T1 values were clipped to a range of 0–2500 ms to suppress background noise and extreme outliers, while post-contrast T1 values were clipped to a range of 0–1200 ms. After intensity clipping, z-score standardization was performed on a per-slice basis. To preserve the quantitative characteristics of the T1 values, histogram equalization, contrast-limited adaptive histogram equalization (CLAHE), and bias-field correction were not applied. All preprocessed images were stored as NumPy arrays and used for subsequent model development and inference.

### 2.3. Model Development

We adopted the SelfRDB architecture [18], a diffusion-based image-to-image translation framework, to generate virtual post-contrast T1 images from native T1 images. The model was developed using the publicly available SelfRDB implementation (https://github.com/icon-lab/SelfRDB (accessed on 2 June 2026)) without introducing additional architectural modules into the original diffusion-bridge framework. To adapt the model for post-contrast T1 synthesis, we modified several hyperparameters, including the input resolution, diffusion schedule, U-Net configuration, and optimization settings. These adaptations are summarized in Table 2 and preserved the core generator–discriminator formulation of SelfRDB.

#### 2.3.1. Diffusion Bridge Formulation

Let $x_{0}$ and y denote a native T1 image and its corresponding post-contrast T1 image, respectively. SelfRDB constructs an interpolating stochastic process as follows:

(1) $$x_{t}=\alpha_{t}x_{0}+\left(1-\alpha_{t}\right)y+\epsilon_{t}$$

where $\alpha_{t}$ ($0\leq \alpha_{t}\leq 1$) follows a monotonically decreasing schedule with respect to t, and $\epsilon_{t}$ represents a Gaussian perturbation whose variance is governed by $\beta_{t}$.

Unlike conventional diffusion bridges, which enforce zero variance at the endpoint, SelfRDB employs a noise schedule in which the variance ($\beta_{t}$) increases monotonically toward the end-point. This design imposes a soft-prior on the source modality by incorporating noise-perturbed source images during training. It thereby relaxes the Dirac-delta constraints, improves model generalizability, and facilitates robust information transfer between the source and target modalities. SelfRDB performs the reverse process using a recovery generator network $G_{\theta }$. The generator recursively produces a self-consistent estimate of the target image, ${\tilde{x}}_{0}^{*}=G_{\theta }(x_{t},\;t,\;y,\;{\tilde{x}}_{0}^{*})$. Using Bayes’ rule and the Markov property, the surrogate estimate ${\tilde{x}}_{0}^{*}$ is then used to compute the posterior transition probability $q(x_{t-1}|x_{t},\;y,\;{\tilde{x}}_{0}^{*})$, from which the intermediate sample $\left(x_{t-1}\right)$ is drawn. This recursive estimation procedure improves sampling accuracy compared with conventional one-shot diffusion approaches [27].

#### 2.3.2. Modifications to the Original SelfRDB Framework

For post-contrast T1 map generation, several hyperparameters were modified relative to those in the default SelfRDB implementation, as summarized in Table 2. The diffusion process consisted of 10 steps with $\beta_{start}=0.1$ and $\beta_{end}=3.0$. These values were selected to limit excessive noise accumulation in the quantitative T1 maps while maintaining stable transitions over the typical T1 range of 200–2000 ms. Recursive self-consistency was enabled for a maximum of two cycles (R = 2), with an early-stopping threshold of τ = 0.01. All other diffusion-bridge components followed standard SelfRDB implementation.

#### 2.3.3. Generator

The generator was implemented as a time-conditional encoder–decoder network based on the SelfRDB U-Net backbone. To accommodate the spatial resolution and characteristics of cardiac T1 images, the channel multipliers, residual blocks, and attention layers of the U-Net were configured as specified in Table 2.

#### 2.3.4. Discriminator

To stabilize model training, we used a patch-based discriminator that jointly evaluated consecutive diffusion states ($x_{t},\;x_{t-1}$). The discriminator was configured with two input channels, a base feature width of 32, and a temporal embedding dimension of 256. A gradient penalty with a weight of 0.5 was applied every 10 training steps.

#### 2.3.5. Training Configuration

Model training was conducted on a Linux workstation equipped with a single NVIDIA RTX A6000 GPU with 48 GB of video memory and an Intel Xeon Silver 4410Y CPU with 48 logical cores and 251 GB of system memory. The workstation ran Ubuntu 22.04 LTS and used NVIDIA driver version 550.144.03 with CUDA 12.4 runtime support. All experiments were implemented using PyTorch Lightning version 2.0.9. The diffusion model was trained using 32-bit floating-point precision with a batch size of 8. The generator and discriminator networks were optimized using the Adam optimizer with momentum parameters of β=(0.5, 0.9). The learning rates were set to $1.6\;\times \;10^{-4}$ for the generator and $1.0\;\times \;10^{-4}$ for the discriminator. Training was performed for a maximum of 1000 epochs, and model validation was conducted every 10 epochs. The checkpoint obtained at epoch 999 was selected as the final model because the validation error remained stable during the later stages of training, while the generator loss continued to decrease gradually. The training and validation learning curves are presented in Supplementary Figure S1.

### 2.4. ECV Map Calculation

The trained model generated virtual post-contrast T1 maps, which were combined with the corresponding native T1 maps to calculate virtual ECV maps using the following equation [28].

(2) $$ECV=\left(1-hematocrit\right)\times \frac{\left(\frac{1}{{T1}_{myo,post}}\;-\;\frac{1}{{T1}_{myo,native}}\right)}{\left(\frac{1}{{T1}_{blood,post}}\;-\;\frac{1}{{T1}_{blood,native}}\right)}\times 100$$

where ${T1}_{myo,post}\;$ and ${T1}_{myo,native}$ denote myocardial T1 values measured on the post-contrast and native T1 maps, respectively. Similarly, ${T1}_{blood,post}$ and ${T1}_{blood,native}$ denote T1 values measured within the LV blood pool on the post-contrast and native T1 maps, respectively. For virtual ECV map calculation, $\;{T1}_{myo,post}$ and ${T1}_{blood,post}$ were obtained from the virtual post-contrast T1 maps. For reference ECV map calculation, the corresponding values were obtained from the acquired post-contrast T1 maps. For each slice, hematocrit was estimated from the native blood-pool T1 value, ${T1}_{blood,native}$, using a synthetic hematocrit model [29]. The same slice-specific hematocrit estimate was used to calculate both the reference and virtual ECV values.

### 2.5. Evaluation

The quantitative T1 values obtained from the AI-generated virtual post-contrast T1 maps and the actual reference post-contrast T1 maps were combined to evaluate the model performance. To ensure anatomically consistent region-of-interest (ROI) measurements, predefined automated masks of the myocardium and LV blood pool were utilized. For each slice, pixel-wise T1 relaxation times were extracted from the myocardial and blood pool ROIs. The mean myocardial and blood-pool post-contrast T1 values were then calculated for both the virtual and actual post-contrast T1 maps.

The image quality of the virtual post-contrast T1 maps was quantitatively evaluated using the peak signal-to-noise ratio (PSNR) and structural similarity index measure (SSIM). These metrics were calculated between the reference and virtual post-contrast T1 maps in the held-out test set after each image had been normalized to the range of 0–1. The resulting image-quality metrics were also compared with those reported in the original SelfRDB study [18].

To evaluate the accuracy and quantitative agreement of the virtual ECV maps, ECV values derived from the AI-generated post-contrast T1 maps were compared with those derived from the actual post-contrast T1 maps. Agreement between the reference and virtual ECV values was assessed using the root mean square error (RMSE) and coefficient of determination (R^2^). Linear regression analysis was performed to characterize the relationship between the reference and virtual measurements.

Bland–Altman analysis was conducted to quantify systematic bias and the limits of agreement (LoA) between the reference and virtual ECV measurements. The mean difference, representing the bias, and the 95% LoA, defined as the bias ± 1.96 standard deviations of the differences, were calculated.

Because the native and post-contrast T1 scans were acquired separately and were not reliably spatially co-registered, voxel-wise comparison of myocardial ECV values was not performed. Instead, myocardial ROIs were independently obtained from the native and post-contrast T1 maps, and a mean myocardial ECV value was calculated for each slice. The resulting slice-level pairs (${ECV}_{ref,\;i}$, ${ECV}_{virt,\;i}$) were matched according to slice identity (i.e., the same slice index) rather than by voxel-wise correspondence. A paired two-sided *t*-test was performed using the slice-level mean ECV differences, calculated as virtual minus reference ECV, to determine whether the mean paired difference differed significantly from zero. Because the absence of a statistically significant difference does not demonstrate clinical equivalence, a two one-sided tests (TOST) procedure was additionally performed using the same slice-level mean ECV values. A prespecified equivalence margin of ±3 percentage points of ECV was used, based on clinically meaningful thresholds reported in previous CMR studies [30]. Statistical equivalence was concluded when both one-sided tests were significant at a significance level of α = 0.05.

## 3. Results

### 3.1. ECV Prediction

**Figure 2:**
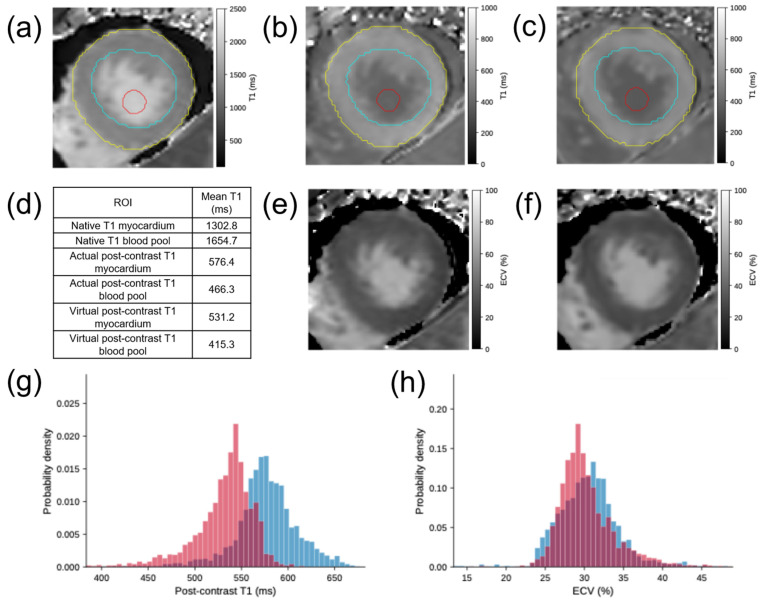
A representative example of myocardial post-contrast T1 and ECV distributions. (**a**) Native T1 map. (**b**) Actual post-contrast T1 map. (**c**) Virtual post-contrast T1 map. The myocardial ROIs are the regions surrounded by the epicardial (yellow) and endocardial (cyan) contours, while the blood pool ROIs are enclosed by the red circles. (**d**) A table of mean T1 values in individual ROIs. (**e**) Actual (or reference) ECV map. (**f**) Virtual ECV map. (**g**) Histograms of T1 values in the myocardium ROIs of the actual and virtual post-contrast T1 maps. A distribution of the myocardium ROI of the actual post-contrast T1 map is indicated by blue color, while that of the virtual post-contrast T1 map is indicated by red color. (**h**) Histograms of T1 values in the myocardium ROIs of the actual and virtual ECV maps. A distribution of the myocardium ROI of the actual ECV map is indicated by blue color, while that of the virtual post-contrast T1 map is indicated by red color.

Virtual post-contrast T1 maps were compared with the actual post-contrast T1 maps within the myocardial regions. The corresponding histograms showed similar overall distributions, although noticeable shifts in their peaks were observed. For instance, in the case shown in Figure 2, the mean myocardial T1 value was 531.2 ms on the virtual post-contrast T1 map, compared with 576.4 ms on the actual post-contrast T1 map (Figure 2d). After transformation to ECV, however, the mean myocardial ECV value derived from the virtual post-contrast T1 map was 30.42% (Figure 2f), closely matching the reference value of 30.32% (Figure 2e).

In the held-out test set comprising 84 slices, the estimated hematocrit had a mean value of 0.51, a standard deviation of 0.05, and a range of 0.40–0.67.

**Figure 3:**
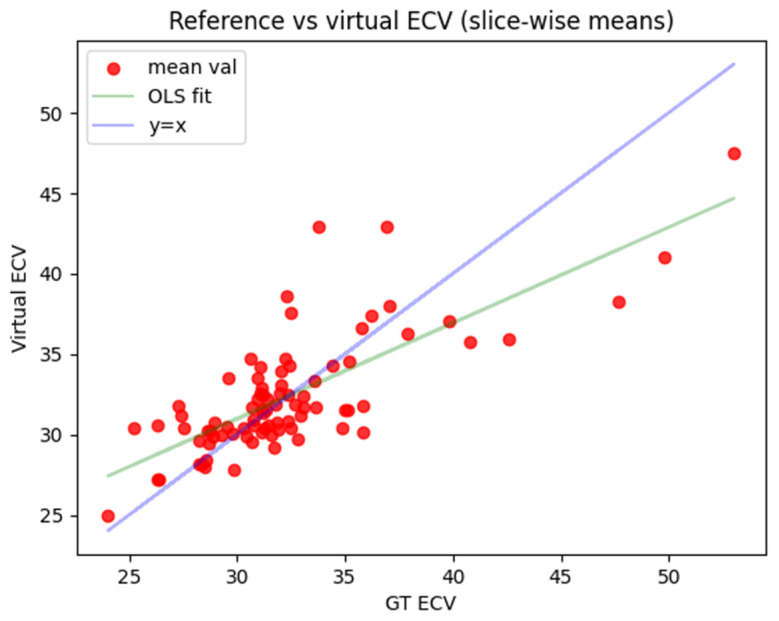
The scatter plot of the mean myocardial ECV values. The ‘mean val’ implies the ECV averaged over the entire myocardial region of interest. The green colored ‘slope’ is a regression line estimated using the ordinary least squares fitting on the samples (red color).

The RMSE between the slice-level mean myocardial ECV values obtained from the reference and virtual ECV maps was 3.05%. The virtual ECV values were strongly correlated with the actual reference ECV values (Pearson r = 0.765; 95% confidence interval [CI], 0.659–0.841; *p* < 0.0001), with a coefficient of determination of R^2^ = 0.585 (Figure 3).

**Figure 4:**
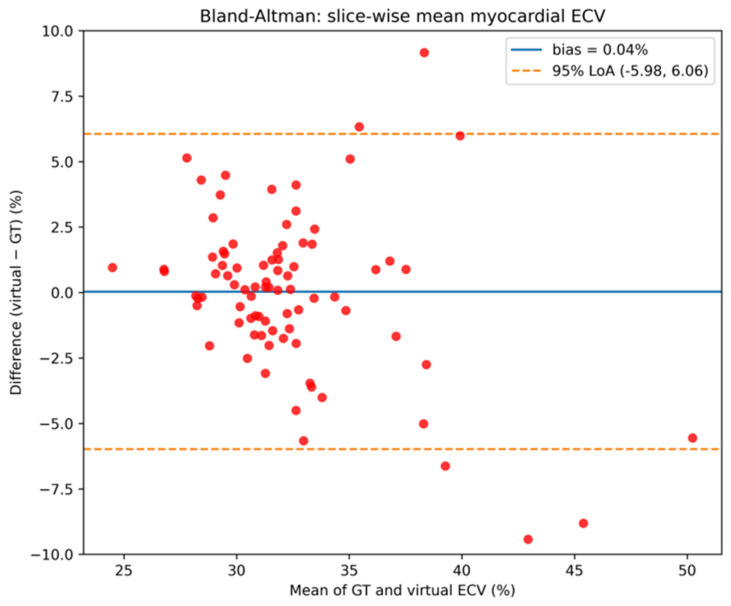
The Bland–Altman plot of the ECV values. Abbreviations: GT, ground truth; ECV, extracellular volume fraction; LoA, limits of agreement.

Bland–Altman analysis revealed minimal systematic bias, with a mean difference of 0.036% between the virtual and reference ECV values (Figure 4). The standard deviation of the paired differences was 3.07%, resulting in 95% LoA of −5.98% to 6.06% (Figure 4). The near-zero mean bias indicated no systematic overestimation or underestimation of reference ECV at the group level. However, the width of the 95% LoA indicated noticeable slice-to-slice variability.

The paired two-sided *t*-test of the slice-level mean ECV differences was not statistically significant (t = 0.107, *p* = 0.915), providing no evidence of a systematic mean bias. The TOST procedure yielded a mean bias of 0.036%, with a 90% CI of −0.52% to 0.59%, and demonstrated statistical equivalence within the prespecified margin of ±3 percentage points (*p* < 0.001).

### 3.2. Virtual Post-Contrast T1 Image Quality

In the held-out test set of 84 slices from 28 patients, the proposed model achieved a PSNR of 22.95 ± 1.77 dB and an SSIM of 82.28 ± 1.87% for virtual post-contrast T1 image generation. These values were lower than those reported for the original SelfRDB framework in brain MRI image-to-image translation tasks. For example, SelfRDB achieved a PSNR of 28.37 ± 1.60 dB and an SSIM of 93.66 ± 2.00% for T2-to-T1 translation using the BraTS dataset and a PSNR of 31.05 ± 1.27 dB and an SSIM of 95.69 ± 0.99% for proton-density-to-T1 translation using the IXI dataset.

In addition, we compared the image quality obtained using modified SelfRDB configuration with that obtained using a configuration closer to the original SelfRDB settings on the validation set. As shown in Supplementary Table S1, the configuration using an image matrix of 128 × 128 and a batch size of 4 yielded a PSNR of 17.32 dB, an SSIM of 48.9%, and a mean squared error (MSE) of 0.111. In comparison, the modified configuration using an image matrix of 224 × 224 and a batch size of 8 achieved a PSNR of 21.67 dB, an SSIM of 82.4%, and an MSE of 0.033.

**Figure 5:**
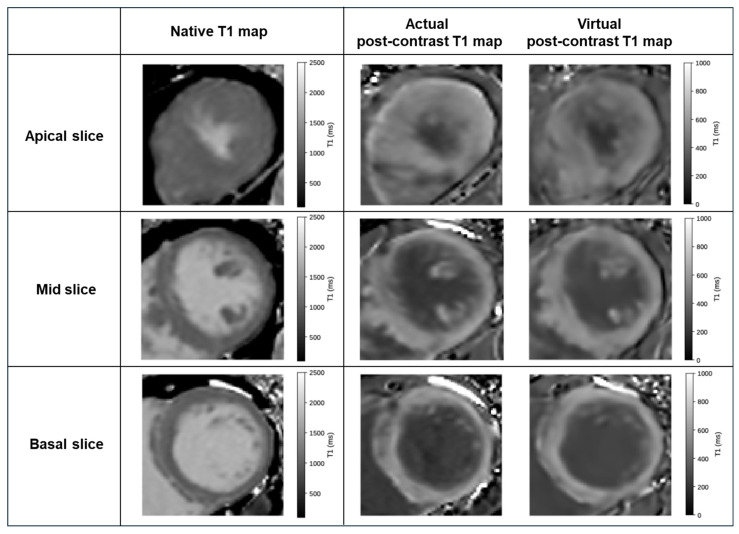
A comparison of image quality between actual post-contrast T1 and virtual post-contrast T1 maps. The image quality comparison indicates that actual and virtual post-contrast T1 maps appear similar.

**Figure 6:**
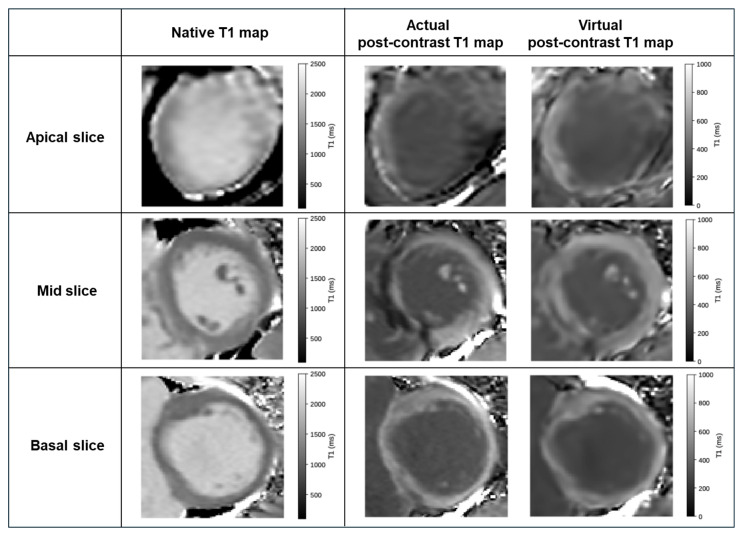
A comparison of image quality between actual post-contrast T1 and virtual post-contrast T1 maps. The image quality comparison indicates that the virtual post-contrast T1 map in the midventricular slice lacks in visualizing dark appearance in the myocardial septum.

Figure 5 presents representative native T1 maps, actual post-contrast T1 maps, and AI-generated virtual post-contrast T1 maps at the apical, midventricular, and basal short-axis levels. Overall, the virtual post-contrast T1 maps showed visual characteristics similar to those of the actual post-contrast T1 maps. In the basal slice, both images showed a similar regional pattern of relatively altered myocardial signal in the septal region. Figure 6 presents a representative case with suboptimal virtual post-contrast T1 image quality. The discrepancy was most evident in the midventricular slice, in which the myocardial septum appeared brighter on the virtual post-contrast T1 map than on the actual post-contrast T1 map.

### 3.3. Computational Time

All training and inference benchmarks were measured on a local workstation equipped with a single NVIDIA RTX A6000 GPU. The mean training time was approximately 73.5 s per epoch. Training for 1000 epochs required approximately 20.4 h in total.

During inference, the end-to-end processing time was approximately 1.41 s (1412 ms) per slice. The mean processing time for each stage was as follows:

- Image preprocessing, including loading the native T1 map and corresponding masks, tensor preparation, resizing or padding to 224 × 224 pixels, and input normalization, required approximately 106.6 ms per slice.
- Virtual post-contrast T1 map generation using the trained SelfRDB model required approximately 1305 ± 41 ms per slice.
- Vectorized calculation of the virtual ECV map from the predicted post-contrast and native T1 values required approximately 0.65 ms per slice.

Overall, the proposed framework generated virtual post-contrast T1 and ECV maps in less than 1.5 s per slice.

## 4. Discussion

This study demonstrates the feasibility of a modified Self-consistent Recursive Diffusion Bridge (SelfRDB) model for generating virtual post-contrast T1 maps directly from native T1 maps without gadolinium-based contrast agent administration. The main finding is that clinically relevant ECV measurements can be derived from contrast-free native T1 maps through AI-generated virtual post-contrast T1 maps.

Slice-wise mean virtual ECV values were in closer agreement with the reference ECV values than the virtual post-contrast T1 values were in with the reference post-contrast T1 values. This behavior can be explained by both the formulation of the ECV calculation and the characteristics of the virtual post-contrast T1 maps. For each slice, the myocardial and blood-pool post-contrast T1 values used in Equation (2) are derived from the same AI-generated post-contrast T1 map. Consequently, although the virtual post-contrast T1 map may exhibit a systematic offset in absolute T1 values (ms), it is expected to preserve the relative contrast between the myocardium and blood pool because both tissue compartments are generated by the same prediction model. Since ECV is calculated as a normalized ratio of relaxation-rate changes (R_1_ = 1/T_1_) between the native and post-contrast states, systematic offsets that similarly affect both the myocardial and blood-pool post-contrast T1 values are partially compensated during the ratio calculation rather than being directly propagated to the final ECV values. As a result, discrepancies observed in the virtual post-contrast T1 maps are attenuated after ECV transformation, leading to closer agreement between the virtual and reference ECV measurements.

In the held-out test cohort, myocardial and blood-pool post-contrast T1 errors were strongly correlated (Pearson r = 0.83), with errors occurring in the same direction in 85% of the slices. The mean biases were −7.6 ms and −4.5 ms for the myocardium and blood pool, respectively. In contrast, the slice-wise mean ECV bias was only 0.04% (SD, 3.1%). For example, as illustrated in Figure 2, the myocardial post-contrast T1 offset of 45.2 ms (531.2 vs. 576.4 ms) resulted in an ECV difference in only 0.10 percentage points (30.42% vs. 30.32%). These findings support the application of Equation (2) to virtual post-contrast T1 maps, suggesting that correlated prediction errors in the myocardium and blood pool are partially compensated during ECV calculation. Although the slice-wise mean ECV bias was near zero, the 95% LoA (−5.98% to +6.06% ECV) indicates non-negligible slice-to-slice disagreement. This range is comparable to that reported in the previous study [14], where the 95% LoA ranged from −7.00% to 7.47%. This suggests that the residual slice-level errors may affect diagnosis when the ECV estimates are close to a clinical ECV borderline.

A major strength of the proposed framework is its ability to learn the relationship between paired native and post-contrast T1 maps despite imperfect spatial alignment. It is well known that the native T1 maps are difficult to perfectly align with their corresponding post-contrast T1 maps, mainly due to the potential movement of the patient’s heart in the two separate MRI scan sessions. Our approach was based on automatic detection and cropping of the LV and myocardium ROIs, which helped substantially reduce the mis-alignment between the native and post-contrast T1 images. The correspondence between the predicted and reference ECV values suggests that the image translation model learned the relationship between native and post-contrast T1 maps even without the perfect alignment between the training dataset pairs.

For generative image-to-image translation studies, PSNR and SSIM are commonly reported metrics alongside task-specific clinical metrics. Our method produced lower PSNR and SSIM when compared to the original SelfRDB study. It should be noted that the native and post-contrast T1 maps were acquired before and after contrast in separate sessions, the heart is a moving organ, and accurate co-registration is not easily achievable in this setting, unlike the original SelfRDB which was applied to the brain MRI where co-registration is relatively easier to perform than cardiac MRI. Our model was trained on such imperfectly aligned pairs by design. Accordingly, quantitative agreement in ECV rather than image fidelity was considered the primary endpoint of this study.

Synthetic LGE images can be generated retrospectively through mathematical calculations based on actual post-contrast T1 maps [31,32,33,34]. The same principle can be applied to the AI-synthesized virtual post-contrast T1 maps to generate virtual synthetic LGE images. However, a dedicated clinical reader study will be required to evaluate the agreement between synthetic and virtual synthetic LGE images, including inter-observer variability and their potential clinical interchangeability.

Our studies have several limitations. First, the data used in our study were limited to the patients with hypertrophic cardiomyopathy, but our methods can be extended to other disease types such as cardiac amyloidosis, acute myocardial infarction, and myocarditis. Second, several synthetic post-contrast T1 maps generated by the proposed model were subject to failure modes and thus often failed to visualize the lesions in data from patients with infarcts. Third, the method requires accurate epicardial and endocardial segmentation for accurate ECV prediction, and atypical cardiac geometries (e.g., severely hypertrophied myocardium or large anterolateral infarcts) are challenging to accurately segment. Fourth, the numbers of training, validation, and test data were limited to 271 patients. Increasing the number of data samples and validating the performance of ECV predictions would be necessary. Finally, multivendor and multicenter studies were not performed. Future multicenter studies involving multiple MRI vendors are warranted to establish the generalizability of the proposed framework.

## 5. Conclusions

In this study, we developed a modified SelfRDB framework capable of generating virtual post-contrast T1 maps directly from native T1 maps without gadolinium-based contrast agent administration in cardiac MRI. Although systematic offsets were observed in the predicted post-contrast T1 values, transformation into ECV substantially reduced these errors, resulting in minimal bias with respect to the reference ECV despite residual slice-to-slice variability. The proposed framework provides a fully automated workflow for virtual post-contrast T1 and ECV map generation with an inference time of less than 1.5 s per slice. Although external validation and prospective clinical studies are required before clinical adoption, the proposed method represents a promising non-invasive approach for quantitative myocardial tissue characterization, with the potential to reduce examination costs and improve patient safety.

## Figures and Tables

**Table 1:** Numbers of patients and image slices in the training, validation, and test sets.

	Training	Validation	Test	Total
Number of patients	218	25	28	271
Number of slices	654	75	84	813

**Table 2:** Hyperparameters used for model training.

Component	Parameter	Value/Description
Diffusion process	Number of steps	10
	Noise schedule	*β*_start_ = 0.1, *β*_end_ = 3.0
	Recursive self-consistency	Up to two cycles (R = 2); threshold, τ=0.01
Generator	Input channels/resolution	2 (native T1 map and diffusion noise)/224 × 224
	Channel multipliers	[1, 1, 2, 2, 4, 4]
	Residual blocks	Two blocks per resolution scale; BigGAN-style blocks with skip rescaling
	Attention layers	Applied only at a resolution of 16 × 16
	Latent embedding	256 dimensions; three-layer multilayer perceptron
	Activations	Centered input activation function enabled; hyperbolic tangent

## Data Availability

The data presented in this study are available on request from the corresponding author. The data are not publicly available due to privacy restrictions.

## Supplementary Materials

The following supporting information can be downloaded at https://www.mdpi.com/article/10.3390/diagnostics16152308/s1, Figure S1: Training monitoring for the SelfRDB native-to-post T1 model (1000 epochs; validation every 10 epochs). (A) Generator training losses. (B) Validation mean squared error (MSE); early-epoch variability decreased after 300 epochs, with late-training values plateauing near 0.033–0.035 (vertical dashed line: epoch 999 checkpoint); Table S1: Pilot experiments on validation data.

## Abbreviations

The following abbreviations are used in this manuscript:

LGE	Late gadolinium enhancement
ECV	Extracellular volume
CMR	Cardiovascular magnetic resonance
SelfRDB	Self-consistent recursive diffusion bridge
MOLLI	Modified Look-Locker inversion recovery
GAN	Generative adversarial network
TR	Repetition time
TE	Echo time
LV	Left ventricle
ROI	Region of interest
SD	Standard deviation
RMSE	Root mean square error
LoA	Limits of agreement
PSNR	Peak signal-to-noise ratio
SSIM	Structural similarity index measure
