# Supplementary material for: AI-Driven Generation of Post-Contrast T1 and ECV Maps from Native T1 Map in Cardiac MRI
Source: Diagnostics (Basel). 2026 Jul 23;16(15):2308. doi: 10.3390/diagnostics16152308 (PMC13465067; doi:10.3390/diagnostics16152308)
Supplement: Supplementary file 1 [file diagnostics-16-02308-s001.zip › diagnostics-4385570-supplementary.pdf]

Supplementary Material

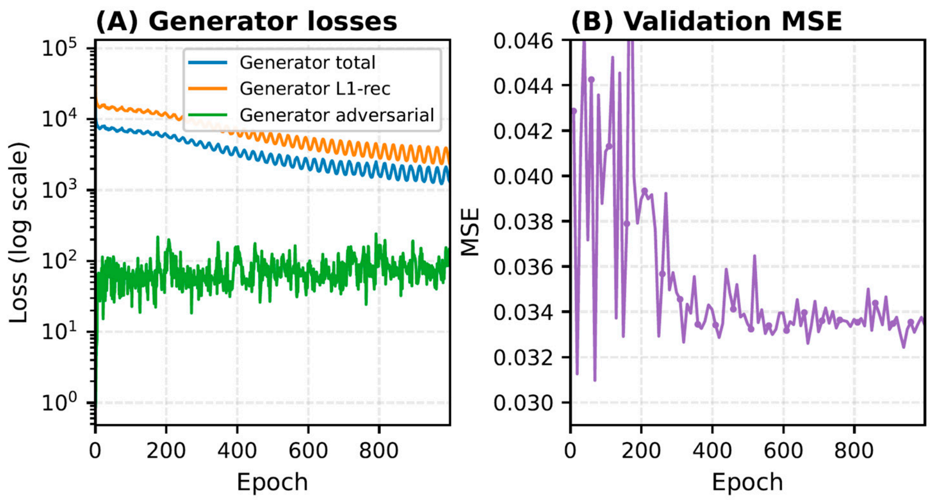

Supplementary Figure S1. Training monitoring for the SelfRDB native-to-post T1 model (1,000 epochs; validation every 10 epochs). (A) Generator training losses. (B) Validation mean squared error (MSE); early-epoch variability decreased after 300 epochs, with late-training values plateauing near 0.033–0.035 (vertical dashed line: epoch 999 checkpoint).

Supplementary Table S1. Pilot experiments on validation data

Model Setting	Image size	Batch size	Number of validation data	PSNR (dB) ↑	SSIM (%) ↑	MSE ↓
Original SelfRDB	128 x 128	4	75 slices	17.32	48.9	0.111
Modified SelfRDB	224 x 224	8	75 slices	21.67	82.4	0.033
